# Perinatal palliative care in sub-Saharan Africa: recommendations for practice, future research, and guideline development

**DOI:** 10.3389/fped.2023.1217209

**Published:** 2023-06-26

**Authors:** Mahlet Abayneh, Sharla Rent, Peter Odion Ubuane, Brian S. Carter, Solomie Jebessa Deribessa, Betelehem B. Kassa, Atnafu Mekonnen Tekleab, Stephanie K. Kukora

**Affiliations:** ^1^ Department of Pediatrics and Child Health, St Paul’s Hospital Millennium Medical College, Addis Ababa, Ethiopia; ^2^ Duke Department of Pediatrics, Duke School of Medicine, Durham, NC, United States; ^3^ Duke Global Health Institute, Durham, NC, United States; ^4^ Department of Pediatrics, Lagos State University Teaching Hospital (LASUTH), Ikeja, Lagos, Nigeria; ^5^ Division of Neonatology and Bioethics Center, Children’s Mercy Hospital, Kansas City, MO, United States; ^6^ Department of Pediatrics and Department of Medical Humanities and Bioethics, University of Missouri-Kansas City School of Medicine, Kansas City, MO, United States; ^7^ College of Health Sciences, Addis Ababa University, Addis Ababa, Ethiopia

**Keywords:** perinatal palliative care, sub-saharan Africa, neonatal intensive care, newborn bereavement, neonatal end-of-life care, low-middle-income-countries

## Abstract

Worldwide, sub-Saharan Africa has the highest burden of global neonatal mortality (43%) and neonatal mortality rate (NMR): 27 deaths per 1,000 live births. The WHO recognizes palliative care (PC) as an integral, yet underutilized, component of perinatal care for pregnancies at risk of stillbirth or early neonatal death, and for neonates with severe prematurity, birth trauma or congenital anomalies. Despite bearing a disproportionate burden of neonatal mortality, many strategies to care for dying newborns and support their families employed in high-income countries (HICs) are not available in low-and-middle-income countries (LMICs). Many institutions and professional societies in LMICs lack guidelines or recommendations to standardize care, and existing guidelines may have limited adherence due to lack of space, equipment, supplies, trained professionals, and high patient load. In this narrative review, we compare perinatal/neonatal PC in HICs and LMICs in sub-Saharan Africa to identify key areas for future, research-informed, interventions that might be tailored to the local sociocultural contexts and propose actionable recommendations for these resource-deprived environments that may support clinical care and inform future professional guideline development.

## Introduction

The death of a child is one of the most devastating human experiences, often resulting in profoundly negative long-term physical and psychosocial consequences on parents (1–3). Stillbirth and early neonatal death is uniquely a loss of both the physical and the envisioned life of the infant (4). The consequent maternal grief typically occurs in private, and is often under-recognized or ignored by the community (5).

Worldwide, more than 2.4 million neonatal deaths (6) and 2.6 million stillbirths occur annually, 98% of which are in low-and-middle-income countries (LMICs) (7). Though neonatal mortality worldwide has substantially improved over the past two decades, it has lagged behind those achieved in under-five children; neonatal mortality still contributes 47% of under-five mortality (6, 7).

In the United States, nearly half (46%) of all deaths in children under 19 years old occur in the first year of life, and two-thirds (66%) of infant deaths occur in the neonatal period (8, 9). Worldwide, sub-Saharan Africa has the highest burden of global neonatal mortality (43%) and neonatal mortality rate (NMR): 27 deaths per 1,000 live births (6). The World Health Organization's Every Newborn Action Plan goal is to achieve a NMR below 12 deaths per 1,000 live births by 2030 (6), but, thus far, only 4/48 (8%) countries have achieved such (10).

Palliative care (PC) is the multidisciplinary prevention or amelioration of pain and other physical, psychosocial or spiritual problems in patients with life-limiting or life-altering conditions and their families with the aim of improving quality of life (11). The WHO recognizes PC as an integral, yet underutilized, component of perinatal care for pregnancies at risk of stillbirth or early neonatal death, and for neonates with severe prematurity, birth trauma or congenital anomalies in both high-income countries (HICs) and LMICs (12). Despite bearing a disproportionate burden of neonatal mortality, many strategies to care for dying newborns and support their families employed in HICs are not available in LMICs. Likewise, many institutions and professional societies in LMICs lack guidelines or recommendations to standardize care, and existing guidelines may have limited adherence due to lack of space, equipment, supplies, trained professionals, and high patient load (13). Gaps in provision of newborn intensive care and opportunities for improvement have been identified in the literature previously (13–16). In this narrative review, we compare perinatal/neonatal PC in HICs and LMICs in sub-Saharan Africa to identify key areas for future, research-informed, interventions that might be tailored to the local sociocultural contexts and propose actionable recommendations for these resource-deprived environments that may support clinical care and inform future professional guideline development.

### Neonatal end-of-life care in high income countries

In HICs, it is recommended that compassionate care for patients with life-limiting or life-altering conditions should begin as soon as a relevant diagnosis is made, including prenatally (17, 18). However, referral for prenatal/neonatal PC counseling, or bereavement counselling following perinatal loss remains variable (19–22). Even the training of neonatologists in end-of-life (EOL) care provision remains variable (23).

Approaches to EOL care for newborns have been empirically studied in the United States and other HICs. These data increasingly inform the creation of standardized care guidelines, policies, and educational opportunities (24), to ensure high-quality care is delivered to each patient while also addressing the unique needs of every patient and family (25). Findings from this research highlight the importance of timely, clear, and empathetic communication and decision-making (26, 27); supporting good parent beliefs (18, 28–31); privacy and memory-making (30, 32, 33); symptom management (29, 34–37); bereavement (30, 38, 39); and the role of palliative care subspecialists (24) and mental health professionals (30, 40–42) (Table 1).

**Table 1:** Summary of elements of high quality perinatal/neonatal palliative care in high income countries.

Timely, Clear, Empathetic Communication	• Sensitive and compassionate language supports families’ trust in the care team (26). • A shared decision-making approach to goals-of-care decisions is recommended (27). • The shared decisional model avoids placing the full burden of responsibility for EOL decision-making on parents. • Parents may choose the degree of engagement and participation in the decision-making process based on cultural norms, but allowing them to have a voice supports their historic, sociocultural, and legal authority to make decisions aligning with their values.
Good Parent Beliefs	• Feeling like they were “good parents” is a crucial for EOL decision-making and subsequent coping (28). • Practices that emphasize the parental role and family-centered care creates positive memories, loving, and affirming parental experiences (29). • Honoring family wishes around the time of death supports positive memory-making (18, 30, 31).
Privacy and Memory Making	• Private rooms or spaces allow families to spend time with their child at EOL. • Music therapists, child life specialists, and spiritual care services are recommended (32, 33). • The care team can facilitate music and appropriate rituals, and aid in creating mementos such as foot/handprints or plaster moldings, photography, or heartbeat recording (30).
Symptom Management	• Parents who feel their newborn suffered pain or distress experience higher levels of post-traumatic stress disorder (PTSD) and prolonged grief (34). • Symptom management and anticipatory guidance for parents about the dying process are critical. • Standardized pharmacologic dosing and administration strategies for mitigating neonatal pain and agitation avoids underutilization of medication (for fear of hastening the patient's death) (35). • Anticipatory planning around discontinuing enteral and parenteral nutrition (36) or withdrawing life-sustaining medical technology (LSMT; e.g., compassionate extubation), may mitigate physiologic signs of the dying process (29). • When withdrawal of supportive technologies and therapies is ethically permissible, yet families may feel troubled; clinicians must clarify that LMST is no longer a treatment but solely an intervention (37).
Bereavement	• Bereavement team members can also be instrumental in supporting grieving families (30). • Specialized staff may assist families and offer psychosocial support during EOL and bereavement (30, 38, 39). • Individual EOL care champions or committees may serve the NICU well in providing excellent EOL care at the bedside and in developing unit-specific best practices for supporting distressed families.
Palliative Care	• Palliative care subspecialists and interprofessional palliative care teams often compliment neonatal staff in communicating with families and supporting goals-of-care decision-making antenatally and throughout the neonate's course. • These clinicians may assist in developing a birth plan consistent with parental wishes, providing ongoing support, and arranging hospice care at discharge. • Hospice or palliative care at home focuses on the baby's comfort or provides continuity for those with complex medical care needs in the face of a life-limiting condition (24).
Mental Health Professionals	• Mental health professionals are helpful in meeting the emotional needs of bereaved parents and developing coping strategies and support networks (40). • In-person, telephonic, or email peer-to-peer parent support may also support parents’ • Individual family members or the broader community may also facilitate support groups to assist parents over the longer term (41). • To counter feeling lonely or abandoned after leaving the hospital (42), some parents find comfort in maintaining connections to staff members who cared for their baby while they were alive (30).

### Neonatal end-of-life care in low- and middle-income countries in sub-Saharan Africa

Though pediatric PC in LIMCs has witnessed some growth in the past several years, this has largely focused on supporting EOL care for older children with conditions like human immunodeficiency virus (HIV) and malignancies (15, 43–45). Perinatal-Neonatal PC is poorly described in global resource-constrained settings, with the limited existing literature focused on how cultural and structural factors limit support of mothers who have suffered stillbirths (46–49). In LMICs, differences in the socio-cultural, religious/spiritual and legal environment, with limited and inconsistently available resources limit the applicability of evidence-based approaches derived in HICs. However, there are actionable steps that may address PC needs, improve bedside care and provide opportunity to develop research-informed interventions (50).

#### Communication around serious news

Physicians practicing in LMICs are faced with considerable challenges and barriers to communicating serious news to parents. Language and cultural barriers impact communication around EOL and bereavement care in sub-Saharan Africa, partly because multiple languages/dialects are spoken in most countries (51–54). Differences in education level, health literacy, and health beliefs between clinicians and parents/families also create challenges in clinical care, particularly in settings where trained medical interpreters do not exist (55, 56). For example, the local word used for “Neonatal Intensive Care Unit (NICU)” may not convey that it is an intensive care unit, making it hard for parents to conceptualize the care provided (56). Cultural norms also impede parents' and families' willingness to express that they do not understand medical information being explained to them or to ask questions of healthcare professionals.

Unlike HICs, where ultrasonography, genetic testing, and other prenatal evaluations may identify which newborns are likely to be imperiled or have a poor prognosis, such anticipation is only recently being provided in LMICs with the expanding use of ultrasound (57). Thus, many perinatal/neonatal deaths happen unexpectedly, without adequate preparation and discussion with the family. Even when critical illness is anticipated, without ample resources or clinician training in communication skills, these deaths may still be complicated and distressing for families. Frequently, the counseling for such scenarios may be done by various individual clinicians in the obstetrical or pediatric setting without the full team of physicians who will be involved in the care. Inconsistent messaging or forecasting may result—confusing parents, creating doubt, or leading to mistrust of the clinicians and their prognostication. For example, if a mother is counseled that her newborn will die immediately after birth, but the baby survives for several days, or longer, it is likely to be distressing for both the mother and the family.

Limited communication undermines parents’ trust and cause families to attribute an infant's death to poor medical care (14, 51, 58). Therefore, clinicians, even while providing clear and consistent messaging about the infant's condition and prognosis, must also acknowledge and explain uncertainties. Currently, there are limited published studies offering insight into preferred phrasing or approaches to these conversations (46). Further research exploring how parents wish to be told difficult news about their newborn's diagnosis and prognosis and how best to engage them in goals-of-care discussions and decision-making are needed.

For physicians-in-training, serious news communication may be especially challenging. Currently, while didactics in ethics may be provided, mentorship and training in communication skills may be inadequate. Clinicians may experience anxiety around difficult conversations and parents may misconstrue the doctor as incompetent. Such discomfort, paired with the time constraints of managing a busy newborn service, may lead the clinician to deprioritize these discussions or even avoid them altogether (46).

Availability of Pediatric PC training is limited in sub-Saharan Africa, therefore physicians lack the support of these subspecialists in complex communication and decision-making. However, models utilizing medical and nursing students, and lay community healthcare workers have been described as stop-gap measures (16). Though the World Health Organization (WHO) explicitly recognizes PC as human right to health (11), funding is needed to develop these capabilities in already over-burdened health systems. National health policies should prioritize developing programs that teach interdisciplinary and interprofessional clinicians, including obstetricians, pediatricians/neonatologists, and nurses, communication approaches and bedside perinatal/neonatal PC skills.

Recommendations:

1. *Once the risk of serious illness and death is identified, clinicians should involve parents/family in discussions about their infant's diagnosis and prognosis as soon as possible. Parental understanding of their infant’s condition, treatments, and care options available–as well as uncertainty and the limits of medicine–should be supported*.
2. *All clinicians, but especially doctors, should communicate clearly with parents/family, preferably in their own language, using clear, culturally sensitive terms that they understand. This may require the counselor to spend extra time to explore parental educational and socio-cultural backgrounds and health literacy*.
3. *Medical Schools and hospitals should provide communication skills training; health workers should be trained periodically in these skills, ideally every 3–5 years*.
4. *Hospitals and professional societies should recognize and encourage motivated health workers who advocate and practice PC*.
5. *National-level health policies should prioritize funding for program development around EOL communication skills and care provision and subspecialty trainings in pediatric PC*.
6. *Perinatal/neonatal PC should be integrated into all existing or new perinatal/neonatal guidelines e.g. Helping Babies Breathe*.

#### Preferences of parents and families for palliative and bereavement care

Due to the varied social, cultural, and resource considerations perinatal palliative care and bereavement interventions designed for HICs may not be applicable for the needs of LMICs (50). Research into parents' preferences in bereavement care, in sub-Saharan Africa is currently limited (46); further investigation is much needed. For example, “memory making”, a cornerstone of perinatal bereavement in HICs (59–62), is less practiced in LMICs (47). HIC studies report that the use of photos, mementos, and spending time sitting with or holding the deceased infant can help create a lasting sense of identity for the deceased (59), mitigate parental psychological distress, and influence the resolution of grief (63, 64). Though evidence-based approaches that are culturally-appropriate and tailored to individual parents in LMICs are sparse, efforts can be made to personalize the end-of-life experience for parents and families. Parents should be asked about any preferences for religious or cultural practices that they desire for their infant, and all reasonable efforts to honor these requests should be made to preserve dignity and respect for the patient and family (13). This could mean engaging religious and traditional leaders considered to be beneficial due to their societal stature, heritage, or clerical stature (47, 50).

Parents in LMICs may hold a wide range of values around the meaningfulness of being present during the death of their newborn. While some parents may align with parents in HICs, where studies suggest that seeing and holding one's deceased baby is helpful (65, 66), others may find holding and caring for their neonate in critical condition traumatizing and painful. They may instead prefer to remember or imagine their child as the beautiful, healthy baby. Parental presence at the death of a newborn is currently contrary to some LMICs normative practice. A Nigerian study revealed that only half of women experiencing stillbirth were allowed to see the body of their infant, and none were given the opportunity to hold the infant, although many would have liked to do so (67). Mothers may be discouraged from holding the child's body or thinking about the baby to shield her from emotional and psychological harm (51, 68). To respect and support the plurality of parents' values and perspectives, they should be offered the opportunity, but not pressured, to be present while their infant passes away and their wishes should be accommodated. Often there are space constraints in the NICU, though if a quiet, calming, private space exists, it should be provided to allow parents the free expression of their emotions. Families choosing to be with their infant as they die may have different preferences about other medical care team members being present with them at this time. If parents so desire, efforts should be made to accommodate this, though the absence of social-workers, peer-parent supporters, and NICU-psychologists may create challenges for busy NICU clinicians. Individual parents may also differ on their preferences for memory-making and bonding, and these preferences may be influenced by cultural norms (47). Opportunities for parents to take photographs of their infants, and, if possible, to retain mementos from the hospitalization such as blankets the baby was wrapped in, clothing or a hat that the child wore may be meaningful and comforting to parents in processing their grief (50). However, care should be taken to ensure that such items are not contaminated with potentially infectious body fluids or fomites.

No studies have investigated parents' perceptions of suffering at end-of-life for newborns in LMICs, though they may have similar experiences to parents in HICs. Cultural adages, such as the Ethiopian saying, “blessed are those who have a comforting death” suggest that, in the absence of evidence, care practices to mitigate symptoms in dying neonates are appropriate. Many premature neonates die due to primary central apnea and progressive hypercapnia and hypoxia, without the subjective feeling of “air hunger,” or visible tachypnea, dyspnea, or agitation (69). For gestationally older newborns or those dying of potentially painful disease processes, sedative or analgesic medications should be provided by intravenous, oral, or intranasal route. These include opiate medications such as morphine, fentanyl, or benzodiazepines such as lorazepam or midazolam (70).

Recommendations:

1. *If a newborn's death seems imminent, clinicians should inquire about parents preferences around EOL care and attempt to honor their wishes*.
2. *Clinicians should ask parents if they wish to be alone with their child at the time of death or if they wish for the medical team or family to be present with them and honor their request*.
3. *Hospitals should devote a space for EOL care and bereaved parents that is quiet, calming, and private, where possible*.
4. *Hospitals should consider investing in interprofessional clinician roles, such as social-workers, peer-parent supporters, or psychologists to support parents through the EOL and bereavement processes*.
5. *Hospitals should foster opportunities for memory-making and offer parents mementos, if desired, of their newborn's time alive in the hospital*.
6. *Healthcare providers are also negatively impacted by perinatal deaths but often neglected in provision of palliative services; thus, they may also benefit from psychosocial support and debriefing*.

#### Impact of social and cultural traditions and stigmas on parental grief and mental health

Death of a newborn or fetus is distressing for the parents, which may be worsened by feelings of isolation. While some cultures may view the death of a newborn as the same as any other person, in many cultures, neonatal loss may be viewed differently than for older children and adults. For this reason, in communities where there are cultural and societal traditions for the loss of older patients, comparatively little may be done for parents during the loss of their newborn. For example, in some cultures within Ethiopia, the death of the newborn, especially less than 3 days old, is not considered as a death of a person (14, 68). Social gatherings, burial ceremonies and religious activities, which may be meaningful to some bereaved parents to feel supported and cope with their loss, may not be done in others. Similarly, bereaved parents who wish to claim their newborns body for formal burial may be stopped by hospitals or extended family members. As the rite of burial may hold symbolic meaning for grief and closure, being inhibited in claiming the newborn's body may heighten parents' distress. Hospital policies which neither obligate nor prohibit parents' taking of the newborn's body, coupled with clinician counseling that normalizes diverse cultural and religious practices, may support parents' preferences around their newborn's death.

Cultural practices intended to protect bereaved parents may inadvertently heighten their trauma. In many cultures, discussing the death of a newborn may be considered taboo, or even dangerous, if that culture endorses beliefs that doing so could cause future losses (47). The hidden nature of stillbirth and neonatal loss is embedded in the social construction of personhood, a phenomenon seen most commonly in regions with high NMR (47, 71–73) Often, unique practices around newborn death exist to protect the woman from emotional and psychological harm (68) and protect her future fertility (51, 68). Likewise, well-meaning family and friends may reassure parents that they will “have another baby” rather than acknowledge the loss of this child (14). While these contrast what might comfort bereaved parents in HICs, few studies of bereaved parents in these settings have explored whether parents in these cultures perceive these practices to be protective or not (46, 47). However, even in cultures where directly discussing the newborn death is discouraged, friends, neighbors, and community members may provide grief support by caring for the bereaved parent, bringing food, offering prayers, and keeping company so that they are not alone. Perinatal PC intervention development efforts must be aware of cultural variations in beliefs and customs, while recognizing that pregnancy is a highly personal experience (46). Further research into how core elements of HIC bereavement care packages can translate to LMICs and what new, culturally tailored, interventions need to be developed to guide practice is warranted (50).

Fathers also experience negative mental health outcomes and financial losses following a perinatal death (46), yet remain an understudied group both in HICs and LMICs***.*** Reviews from HICs describe men as having less intense and less enduring levels of negative psychological outcomes, but a greater likelihood of engaging in compensatory behaviors such as alcohol use (74, 75), In LMICs, men may feel marginalized since their female partner's grief is often more visible, and their grief may be heightened from having restricted opportunity to engage with their newborn in the hospital while alive (13). Other works have shown that while perinatal loss can create conflict in some couples, it can lead to a greater sense of closeness in others (76).

In many LMICs, cultural norms about appropriate male behavior may prevent fathers from openly grieving following the loss of their child (76). This grief suppression can increase the risk of chronic psychological issues (5). Also, healthcare workers may impair parental grieving perinatal deaths if they fail to show adequate empathy or fail to disclose important information such as the (presumed) cause of death (77).

Recommendations:

1. *Health workers should understand and acknowledge the grief of parents who have lost a newborn and support them in the bereavement process*.
2. *Clinicians should explain the cause of death as much as possible, highlighting, if appropriate, that it was not caused by a mistake or misdeed by the mother or father*.
3. *Hospitals and institutions should empower and support parents in their wishes regarding their newborn's body after death so that religious or cultural customs may be honored*.
4. *Hospitals and institutions should create opportunities for ongoing support of bereaved parents through ongoing engagement with caregivers who met their child while alive, similar to bereavement follow-up programs in HICs. They should also engage with local communities to identify and integrate hospital-based bereavement support with culturally-appropriate community-based programs and resources*.

#### Life, death, personhood, and the law

Though there remains an ongoing debate in HICs around the moral status of a fetus and when a fetus achieves personhood (78–80), determining which critically-ill neonates should have intensive therapies offered, mandated, or withheld creates complex, but navigable ethical challenges. Regionalization of neonatal intensive care in HICs promotes justice, ensuring that wherever a newborn is delivered they are entitled to the same care options as at any other center. While disparities in neonatal outcomes remain in the United States (81), these are narrower than outcome disparities between urban and rural settings in sub-Saharan Africa. The availability of population outcome data anchors ethical decision-making; for example, discrete gestational-age based thresholds for offering and obligating resuscitation can be defined for extremely premature delivery. For cases in which population data do not exist, such as for serious congenital anomalies or genetic conditions, frameworks exist for guiding the boundaries of parental discretion (82) and identifying which therapies are impermissible, permissible, and obligatory (83). In HIC, Hospital Ethics Committees may assist if there is conflict between parents and clinicians, or the best interest of the neonate is unclear. Shared decision-making between clinicians and parents is recommended in situations where outcomes are uncertain and the acceptability of the outcomes are based on personal values, such as feelings about quality of life (27, 84). Such considerations may be aspirational in LMICs in view of healthcare capacity, communitarian approaches to decision-making, and cultural norms.

In resource-constrained settings, what is defined as life-limiting or life-altering may be inconsistent across hospitals within the region and country. Instead, a “life-limiting” congenital anomaly with expected early demise is based upon whether the hospital can treat the condition, which can vary from day-to-day within the same hospital based on availability of equipment, supplies, and personnel. Significant disparity in survival remains for extremely premature infants, often referred to as the “90:10 survival gap,” noted between likelihood of survival in HICs:LMICs (85). Over 90% of babies born before 28 weeks gestation survive in HICs but only about 10% of babies in this same gestational age range survive in LMICs. Though outcomes in HICs remain variable at early gestational ages (86–89), guidance from professional organizations bound resuscitation decisions to a somewhat narrow gestational age range (90–92). Regionalization and well-resourced transport systems ensure that critically ill newborns may receive care at centers that are able to meet their needs (93). This is largely not so in many LMICs where there is wide disparity in the resources and expertise for quality care of extreme preterm infants, often necessitating continued adoption of the 28-week cut-off viability age for ethical-legal reasons (94). Frameworks for considering peri-viability resuscitation decision-making in HICs cannot be directly applied in LMICs (95). Often, no national, regional, or institutional guidelines exist to prescribe a standardized approach to these decisions. The WHO defines “late stillbirth” as fetal deaths at ≥1,000 g or ≥28 weeks of completed gestation for the purpose of standardizing data collection for international pregnancy outcome comparison (85). While clinicians practicing in many LMICs may consider 28-weeks as the “official” gestational age below which any infant “should” be considered a stillbirth or miscarriage, the equitable utilization of resources or the determination that a communitarian vs. individualistic notion of the baby's best interests may be unclear (94, 95). Likewise, a paucity of child-protection laws creates ambiguity around where the limits of parental authority lie, and what healthcare decisions for their neonates are “harmful.” Parents are often personally financially responsible for their newborn's care in the hospital, and whether they are able to pay for care may factor into decisions of what therapies are provided (13). These factors necessitate a different approach to ethical reasoning around neonatal resuscitation and end-of-life care decision-making that both supports the best interest of the infant and also respects values held by parents. This is, particularly important in light of cultural feelings around disability stigma and the limited resources available to support the health and well-being of severely impaired survivors. Additional research into where clinicians and parents/families experience ethical challenges in newborn care are needed to guide development of culturally-relevant and context-specific standardized approaches to ensure justice (96). Regionalization and well-resourced transport systems in LMICs may ensure that critically ill newborns may receive care at centers that are able to meet their needs (93, 97), though challenges in implementation remain (13).

Though several sub-Saharan African countries (98) have ratified the African Union's Protocol on the Rights of Women in Africa (99) which requires member-states to “protect the reproductive rights of women by authorizing medical abortions…where the continued pregnancy endangers the mental and physical health of the mother or the life of the mother or the fetus,” (99) abortion laws vary widely among countries (100). In many countries, medically-indicated abortion may be legal after serious fetal diagnosis, although access may still be limited by resource-constraints (101, 102), cultural stigma (103–108), and lack of knowledge and awareness of such provisions amongst both providers or patients (102, 109–111). Complex social/cultural/religious factors also influence abortion decisions, often raising ethical dilemmas and moral distresses among clinicians (112–115). Though studies have investigated how pregnant patients seeking abortion consider these decisions (104, 106, 116–121), few have specifically sought the perspectives of patients who have experienced pregnancies complicated by serious fetal conditions (121). Future research exploring the perspectives of these patients when making decisions around pregnancy termination is needed.

Recommendations:

1. *Professional societies should collaborate with legislators and government agencies to prepare country-wide guidelines to standardize counseling and care provision for neonatal conditions. These guidelines should take into consideration the resources at different levels of the health system and the local transport/referral system available to escalate care if needed*.
2. *Healthcare systems should strive to support regionalization of neonatal care, to ensure that critically ill newborns may be transported to centers matching their care needs*.
3. *Clinicians should identify individual clinical opportunities for parent engagement and shared decision-making when outcomes are uncertain and values-based*.
4. *Hospitals should develop pediatric ethics committees to address situations where neonatal best-interest and the role of clinicians and parents in decision-making is unclear or conflicts arise*.
5. *Clinicians providing perinatal PC to pregnant patients with serious antenatal diagnoses, should be familiar with the local laws regarding abortion to better appropriately guide informed parental choice within the full range of legally-available options, while being sensitive to their socio-cultural and religious preferences. Trauma-informed approaches (122) examining one's own values and biases (123), and practicing cultural humility (124), may help clinicians avoid unduly influencing, pressuring, or stigmatizing patients in these already stressful situations (103, 110, 116)*.

### Integrating palliative care and psychosocial support tools into existing healthcare structures

Considerable gaps remain in the provision of perinatal PC globally, though many of these gaps can be addressed (50). While some approaches to improving this care may be readily available and cost effective (e.g., clinician training), others will require systems-level and policy-level actions. Possibly, some PC elements developed in HIC settings may be translatable to LMICs, while others will require considerable adaptation, and others will remain inappropriate. Empirical and qualitative research exploring clinicians' and parents' perceptions of PC provision in LMICs, and how local culture and resources impact this care, are needed. Likely, new approaches to context-specific perinatal/neonatal PC will be developed as the field grows globally. As neonatal care improves in LMICs, overall mortality may be reduced, but complex decision-making around limitations of therapies may become more common and more difficult, particularly when ICU beds, supportive technologies such as ventilators, or other resources remain constrained. To address these anticipated future changes and challenges, guidelines and recommendations to optimize and standardize current practices of perinatal/neonatal PC and bereavement care for parents are greatly needed, though these should be flexible and re-examined frequently to adapt to future changes.
